# Bile proteomics for differentiation of malignant from benign biliary strictures: a pilot study

**DOI:** 10.1093/gastro/gou066

**Published:** 2014-10-09

**Authors:** Udayakumar Navaneethan, Vennisvasanth Lourdusamy, Preethi GK Venkatesh, Belinda Willard, Madhusudhan R Sanaka, Mansour A Parsi

**Affiliations:** ^1^Department of Gastroenterology and Hepatology, Digestive Disease Institute, Cleveland Clinic, Cleveland, OH, USA and ^2^Proteomics Core Laboratory, Lerner Research Institute, Cleveland Clinic, Cleveland, OH, USA

**Keywords:** pancreatic cancer, proteomics, bile, cholangiocarcinoma

## Abstract

**Background:** Determining the etiology of biliary strictures is challenging, and the sensitivities of the current tests to diagnose them are low. Protein biomarkers in bile, in combination with other tests, may improve sensitivity in diagnosing biliary strictures.

**Objective:** To analyse the differential abundance of proteins in benign and malignant biliary strictures through proteomic analysis of bile.

**Methods:** In this prospective, cross-sectional study, bile was aspirated in 24 patients undergoing endoscopic retrograde cholangiopancreatography (ERCP) including six patients with primary sclerosing cholangitis (PSC), three with cholangiocarcinoma (CCA), ten with pancreatic cancer, and five with benign biliary conditions. Liquid chromatography/mass spectrometry was used to examine the bile for differential abundance of protein biomarkers. The relative abundance of various proteins was compared in the malignant *vs.* benign groups and in CCA *vs.* PSC.

**Results:** The majority of the proteins identified in bile were similar to those of the plasma (plasma proteins) and certain proteins were differentially expressed among the different groups (CCA, pancreatic cancer, PSC or benign). A total of 18 proteins were identified as being more abundant in the malignant group (CCA and pancreatic cancer) than in the benign strictures group, including myeloperoxidase, complement C3, inter-alpha-trypsin inhibitor heavy chain H4, apolipoprotein B-100, and kininogen-1 isoform 2. A total of 30 proteins were identified to be less abundant in the malignant group than in the benign group, including trefoil factor 2, superoxide dismutase [Cu-Zn], kallikrein-1, carboxypeptidase B and trefoil factor 1.

**Conclusions:** Protein biomarkers in bile may differentiate malignant from benign biliary strictures. Larger studies are warranted to validate these observations.

## Introduction

Biliary strictures can be due to benign or malignant causes. Cholangiocarcinoma (CCA) and pancreatic cancer account for the majority of malignant biliary strictures, and are often associated with grave prognosis at the time of diagnosis [1, 2]. Detecting malignancies at an earlier stage is of paramount importance for effective management. Diagnosing malignant strictures at an early stage remains very challenging, especially when there is no image evidence of a mass lesion (indeterminate biliary stricture).

Endoscopic retrograde cholangiopancreatography (ERCP) with brush cytology and endoscopic ultrasound (EUS) with fine-needle aspiration (FNA) are the usual modalities for diagnosing biliary strictures. EUS-FNA is highly sensitive and superior to ERCP cytology in diagnosing malignant biliary strictures associated with pancreatic mass lesions, yet its sensitivity is similar to brush cytology for diagnosing indeterminate strictures in the setting of CCA [3]. Also, the sensitivities of diagnosing malignant lesions through ERCP brushings in the published studies is low [4–6]. Although the addition of Fluorescence in situ hybridization (FISH) to cytology improves the sensitivity rates of ERCP brushings [7–9], the combined sensitivity is still low in detecting malignant strictures. Lower diagnostic sensitivities of the current techniques to detect malignant strictures warrant additional methodologies to improve the diagnosis.

Use of proteomics to detect biomarkers in bile may hold promise in aiding differentiation of malignant from benign biliary strictures [10]. Previous studies have attempted to study bile proteomics to identify peptides that will differentiate malignant from benign biliary strictures [11–15]. Most of the studies are limited because of lack of co-existing clinical information and a small sample size. The main aim of our study was to analyse the differential abundance of proteins in benign *vs.* malignant biliary strictures.

## Methods

### Patients

The Cleveland Clinic biliary fluid database is a prospectively maintained database of bile obtained by direct aspiration from the common bile duct in patients referred to our center for ERCP. We established this database in 2012 and included all patients in our center who had bile aspirated prior to contrast injection at the time of ERCP. The study was approved by the Cleveland Clinic Institutional Review Board and registered with the National Institute of Health (NIH) clinical trials registry. (NCT01565460) The patients included in our study were recruited between September 2012 and November 2012 and had a minimum of 1 year of clinical follow-up. Informed consent was obtained from each patient included in the study. The study protocol conforms to the ethical guidelines of the 1975 Declaration of Helsinki (6th revision, 2008) as reflected in *a priori* approval by the institution's human research committee.

### Inclusion and exclusion criteria

The inclusion criteria were ability to give informed consent and age >18 years. Patients who had acute cholangitis were not included in our biliary fluid database. The diagnosis of pancreatic cancer and CCA was based on tissue diagnosis, either at surgery or on fine needle aspiration on subsequent endoscopic ultrasound on follow-up. Tissue diagnosis was established, based on histology in all patients with cancer in our cohort.

### Biliary fluid sampling procedure

At the time of ERCP, once we had cannulated the common bile duct, approximately 1 to 5 mL of bile was aspirated through the sphincterotome. We transported these bile samples to the laboratory on ice; they were then frozen at –80°C until use.

### Measurement of protein/peptides in bile

Bile samples were fractionated on a sodium dodecyl sulfate polyacrylamide gel electrophoresis (SDS-PAGE) gel. Each sample was divided into three bands and analysed by liquid chromatograph mass spectrometer (LC-MS) or MS. To determine the protein content, dilutions were made to several samples in an attempt to try and equalize the overall amount of protein present in the SDS-PAGE gel. Several gels were run and the bands were cut from each gel. The protein bands were digested according to an in-gel digestion procedure. Briefly, the bands were cut from the gel and washed in 50% ethanol/ 5% acetic acid, alkylated with iodoacetamide and reduced with Dithiothreitol (DTT). All bands were completely digested ‘in-gel' using trypsin, by adding 5 μL trypsin (10 ng/μL) in 50 mmol/L ammonium bicarbonate and incubating overnight at room temperature. The peptides that were formed were extracted from the polyacrylamide in two aliquots of 30 μL 50% acetonitrile with 5% formic acid. These extracts were combined and evaporated to <10 μL in Speedvac (Thermo Fischer Scientific, San Jose, CA, USA) and then suspended in 1% acetic acid to make up a final volume of 30 μL for LC-MS analysis.

The LC-MS system was a Finnigan LTQ-Obitrap Elite hybrid mass spectrometer system. The HPLC column was a Dionex 15 cm × 75 μm. Acclaim Pepmap C18, 2 μm, 100 Å reversed phase capillary chromatography column. The extract was injected in 5 μL volumes and the peptides, eluted from the column by an acetonitrile/0.1% formic acid gradient at a flow rate of 0.25 μL/min, were introduced into the source of the mass spectrometer on-line. The microelectrospray ion source was operated at 2.5 kV. The digest was analysed using the data-dependent multitask capability of the instrument, acquiring full scan mass spectra, to determine peptide molecular weights and product ion spectra to determine amino acid sequence in successive instrument scans.

### Database searching

Tandem mass spectra were extracted by Proteome Discoverer software, version 1.4.1.288. Charge state de-convolution and de-isotoping were not performed. All LC-MS/MS samples were analysed using Mascot (Matrix Science, London, UK; version 2.3.02), Sequest (Thermo Fisher Scientific, San Jose, CA, USA; version 1.4.0.288) and X! Tandem (The GPM, thegpm.org; version CYCLONE (2010.12.01.1)). Mascot, Sequest, and X! Tandem were set up to search the Human Reference Sequence database (33292 entries) assuming the digestion enzyme trypsin, fragment ion mass tolerance of 1.2 Da and a parent ion tolerance of 20 PPM. Carbamidomethyl of cysteine was specified as a fixed modification and oxidation of methionine was specified as a variable modifications.

### Criteria for protein identification

Scaffold software (version Scaffold_4.3.2; Proteome Software Inc., Portland, Oregon, USA) was used to validate LC-MS/MS-based peptide and protein identifications. Peptide identifications were accepted if they could be established at greater than 95.0% probability by the Peptide Prophet algorithm [16]. Protein identifications were accepted if they could be established at greater than 99.0% probability to achieve a false discovery rate (FDR) of less than 1.0% and contained at least two identified peptides. Protein probabilities were assigned by the Protein Prophet algorithm [17]. Proteins that contained similar peptides and could not be differentiated based on LC-MS/MS analysis alone were grouped to satisfy the principles of parsimony.

### Comparison of malignant and benign biliary strictures

The relative abundance of the proteins was compared by using the label-free quantitative method, spectral counting [18]. This method utilizes a data-dependent analysis, which involves an initial mass scan followed by 20 LC-MS/MS scans on the most abundant peptides. The selection of peptide ions for LC-MS/MS analysis was based on the abundance of the peptides and, therefore, the more abundant the protein in the sample the more often peptides from this protein were selected for LC-MS/MS analysis. The relative quantity of these proteins was determined by comparing the number of spectra (termed ‘spectral counts'), used to identify each protein. The numerical values used in the quantification correspond to the normalized spectral abundance factor (NSAF; SC/ΣSC*protein length) [19]. The error observed for the SC measurements is greater for less-abundant proteins than for more-abundant proteins. Because of this, different filtering criteria were used to determine whether proteins are differentially present, based on the overall abundance, as an NSAF ratio.

**Figure 1.**
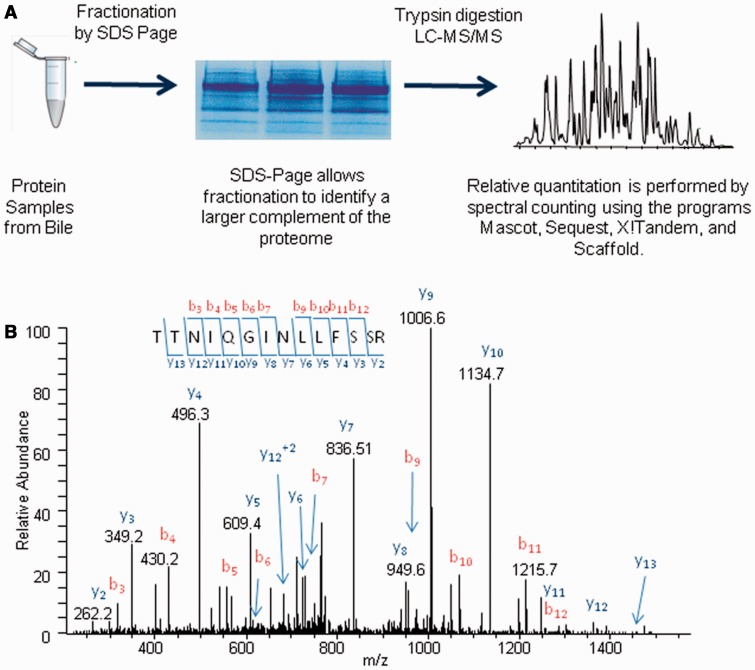
Schematic representation of the proteomic approach used to identify and quantify proteins from bile samples. These experiments involve bile protein isolation, SDS-page fractionation, tryptic digestion, and LC-MS/MS analysis. The resulting data were searched with the programs Mascot, Sequest, and X!Tandem and the proteins were quantified by spectral counts (**A**). An example MS/MS spectrum from the doubly charged 782.4 Da peptide identified in the LC-MS/MS analysis of a cholangiocarcinoma sample. The amino acid sequence of this peptide was identified as TTNIQGINLLFSSR by the presence of several sequence specific N (b) and C-terminal (y) ions. This peptide was matched to the protein complement C4-A isoform 1, which was found to be more abundant in cancer than in control (**B**).

The schematic representation of the proteomics approach to identifying proteins in each bile sample is shown in Figure 1. The error rating of the label-free method used in this analysis is greater for less-abundant proteins and we therefore utilized different filtering criteria for proteins of varying abundance levels (Table 1) [20]. The relative abundances of the proteins in these samples was determined by spectral counts and different cut-off values and *P*-values. Based on the criteria, the relative abundances of 304, 425, and 459 proteins were compared in the CCA *vs.* PSC, and malignant (pancreatic cancer and CCA) *vs.* benign (PSC and benign samples) respectively as set out below.

**Table 1.** Criteria used to determine whether proteins are differentially present

Abundance	SC range	NSAF ratio	*P*-value
Very low	1.7–7	0.4 ≤ NSAF ratio ≥ 2.5	≤0.01
Low	8–19	0.5 ≤ NSAF ratio ≥ 2.0	≤0.01
Medium	20–79	0.5 ≤ NSAF ratio ≥ 2.0	≤0.05
High	80+	0.67 ≤ NSAF ratio ≥ 1.5	≤0.05

SC = spectral count; NSAF = normalized spectral abundance factor

## Results

A total of 24 bile samples were analysed, including six patients with PSC, three with CCA, ten with pancreatic cancer, and five with benign biliary conditions. The patients were recruited from September 2012 to November 2012. Among the five patients with benign biliary conditions, three had sphincter stenosis, one had chronic pancreatitis, and one had benign stricture secondary to biliary stones. All the six patients with PSC had both intrahepatic and extrahepatic PSC. None of the patients had any dominant strictures. Of the three patients with CCA, one had hilar strictures, while the remaining two had distal biliary stricture. All three presented with indeterminate biliary strictures. All patients had a minimum follow-up of at least 1 year up to November 2013. The basic demographic information is summarized in Table 2.

**Table 2.** Demographic and clinical characteristics of study cohort

Variables	Benign biliary conditions (*n* = 5)	Cholangiocarcinoma (*n* = 3)	Pancreatic cancer (*n* = 10)	Primary sclerosing cholangitis (*n* = 6)
Age, mean (SD); years	44.0 (13.7)	78.0 (7.1)	72.1 (8.2)	59.8 (15.0)
Female/male (*n*)	5/0	1/2	3/7	2/4
Albumin, mean (SD); mg/dL	3.3 (1.9)	3.4 (0.7)	3.3 (0.7)	2.6 (2.0)
Bilirubin, mean (SD); mg/dL	0.42 (0.41)	1.3 (0.9)	5.9 (6.4)	1.1 (1.2)
AKP, mean (SD); U/L	66.6 (37.0)	161 (69.1)	505.2 (296.7)	224.3 (214.4)
AST, mean (SD); U/L	26.8 (16.8)	44.7 (23.1)	94.1 (63.4)	42.3 (39.7)
ALT, mean (SD); U/L	28.2 (16.8)	33.0 (4.9)	136.7 (131.9)	49.2 (45.8)
CA 19-9, mean (SD)	NA	85.8 (61.7)	2591.7 (1191.2)	958 (817.3)

AKP = alkaline phosphatase; AST = aspartate aminotransferase; ALT = alanine aminotransferase; NA = not available

Each of the 24 bile samples was subjected to SDS-PAGE fractionation, tryptic digestion and LC-MS/MS analysis for both protein identification and relative protein quantification. Each gel lane was cut into three areas and analysed by a 120-minute LC gradient. The LC-MS/MS experiments identified a total of 459 proteins by at least two peptides. The overall FDR for this analysis was less than 1%.

### Malignant *vs.* benign strictures

A total of 459 proteins were quantified in the malignant and benign groups. The identification of proteins of different abundance was based on the criteria given in Table 1. Eighteen proteins (4%) were identified as more abundant in the cancer samples, including such proteins as MPO, complement C3, inter-alpha-trypsin inhibitor heavy chain H4, apolipoprotein B-100, and kininogen-1 isoform 2. Thirty proteins (7%) were identified as less abundant in cancer and included trefoil factor 2, superoxide dismutase [Cu-Zn], kallikrein-1, carboxypeptidase B and trefoil factor 1. The details were summarized in Tables 3 and 4.

**Table 3.** Proteins identified as more abundant in cancer (malignant strictures) than in controls (primary sclerosing cholangitis and benign strictures)

Proteins	Accession	MW (kDa)	Average SC		NSAF ratio	*P*-value (*t*-test)
			Cancer	Control		
Fibrinogen beta chain isoform 1 pre-pro-protein	70906435	56	138.8	52.1	2.2	0.01481
C4b-binding protein alpha chain precursor	4502503	67	37.3	11.5	2.3	0.04311
Serum albumin pre-pro-protein	4502027	69	986.0	332.8	2.4	0.00006
Vitamin D-binding protein isoform 1 precursor	32483410	53	51.9	19.2	2.4	0.03919
Kininogen-1 isoform 2 precursor	4504893	48	21.3	5.5	3.8	0.01109
Alpha-2-macroglobulin precursor	66932947	163	127.9	29.3	3.9	0.00618
Haptoglobin isoform 1 pre-pro-protein	4826762	45	83.8	18.0	4.2	0.00024
Fibrinogen gamma chain isoform gamma-B precursor	70906439	52	89.3	17.3	4.3	0.00133
Complement C3 precursor	115298678	187	174.7	35.6	4.3	0.00612
Myeloperoxidase precursor	4557759	84	70.2	11.0	5.3	0.02501
Apolipoprotein A-I pre-pro-protein	4557321	31	29.8	5.9	5.7	0.02170
Fibrinogen alpha chain isoform alpha-E pre-pro-protein	4503689	95	63.3	9.3	6.4	0.00031
Alpha-2-HS-glycoprotein pre-pro-protein	156523970	39	7.3	1.1	6.5	0.00173
Ceruloplasmin precursor	4557485	122	47.3	5.6	7.5	0.00114
PREDICTED: complement C4-A isoform 1	341916194	193	32.1	4.5	7.7	0.01791
Alpha-1B-glycoprotein precursor	21071030	54	10.9	1.2	8.3	0.00468
Inter-alpha-trypsin inhibitor heavy chain H4 isoform 1 precursor	31542984	103	5.5	0.2	35.4	0.00484
Apolipoprotein B-100 precursor	105990532	516	49.3	1.0	55.7	0.02595

MW = molecular weight; SC = spectral count; NSAF = normalized spectral abundance factor

**Table 4.** Proteins identified as less abundant in cancer (malignant strictures) than in controls (primary sclerosing cholangitis and benign strictures)

Proteins	Accession	MW (kDa)	Average SC		NSAF ratio	*P*-value (*t*-test)
			Cancer	Control		
Malate dehydrogenase, mitochondrial precursor	21735621	36	0.0	3.5	0.0	0.00897
Prostate stem cell antigen pre-pro-protein	289547757	12	0.0	3.6	0.0	0.00926
Enteropeptidase precursor	223942069	113	0.0	2.9	0.0	0.00964
Kallikrein-1 pre-pro-protein	4504875	29	0.0	10.3	0.0	0.00052
Chymotrypsinogen B2 precursor	118498350	28	0.0	17.6	0.0	0.00086
Colipase isoform 1 pre-pro-protein	4502895	12	0.4	25.9	0.0	0.00009
Chymotrypsin-like elastase family member 3A pre-pro-protein	236460050	29	0.5	29.1	0.0	0.00015
Phospholipase A2 precursor	4505847	16	1.3	39.5	0.0	0.00018
Superoxide dismutase [Cu-Zn]	4507149	16	1.0	15.2	0.0	0.00005
Lithostathine-1-alpha precursor	29725633	19	1.6	30.3	0.1	0.00223
Chymotrypsin-like elastase family member 3B pre-pro-protein	6679625	29	2.4	48.7	0.1	0.00012
Chymotrypsin-C pre-pro-protein	62526043	29	0.9	18.6	0.1	0.00015
Trypsin-1 pre-pro-protein	4506145	27	3.1	41.2	0.1	0.00001
Carboxypeptidase B pre-pro-protein	54607080	47	5.2	79.5	0.1	0.00020
Trypsin-2 pre-pro-protein	4506147	26	2.3	31.7	0.1	0.00412
Trefoil factor 1 precursor	4507451	9	0.7	4.7	0.1	0.00868
Carboxypeptidase A1 precursor	4502997	47	2.7	32.5	0.1	0.00012
Selenium-binding protein 1	16306550	52	2.8	19.1	0.1	0.00339
Pro-activator polypeptide isoform a pre-pro-protein	11386147	58	1.0	7.1	0.1	0.00016
Junction plakoglobin	12056468	82	1.7	13.5	0.1	0.00535
Pancreatic secretory granule membrane major glycoprotein GP2 isoform 2 precursor	119220571	59	4.5	33.7	0.1	0.00291
Desmoplakin isoform I	58530840	332	3.3	20.3	0.1	0.00322
Serpin B6	41152086	43	8.6	40.7	0.2	0.00135
Pancreatic alpha-amylase precursor	4502085	58	8.9	59.9	0.2	0.02459
Trefoil factor 2 precursor	4885629	14	6.2	20.6	0.2	0.00049
Non-secretory ribonuclease precursor	4506549	18	3.6	9.7	0.3	0.00830
Retinol-binding protein 4 precursor	55743122	23	10.5	26.6	0.3	0.00201
Hornerin	57864582	282	7.8	18.7	0.4	0.00530
Antithrombin- III precursor	4502261	53	27.8	44.5	0.5	0.01112

MW = molecular weight; SC = spectral count; NSAF = normalized spectral abundance factor

### PSC *vs.* CCA

A total of 304 proteins were quantified in the CCA and PSC groups. Fourteen proteins (6%) were identified as more abundant in cholangiocarcinoma, including such proteins as intercellular adhesion molecule 1, ceruloplasmin, haptoglobulin, complement c3, and -c4b. Nine proteins (3%) were identified as less abundant in cholangiocarcinoma, which included pro-activator polypeptide isoform a pre-pro-protein, carboxypeptidase B, chymotrypsin-like elastase family member 2A, chymotrypsin like elastase family member 3B, and chymotrypsin-C. The details are summarized in Tables 5 and 6.

**Table 5.** Proteins identified as more abundant in cholangiocarcinoma than in primary sclerosing cholangitis

Proteins	Accession	MW (kDa)	Average SC		NSAF ratio	*P*-value (*t*-test)
			CCA	PSC		
Serum albumin	4502027	69	1338.0	334.5	2.16	0.03297
Alpha-2-macroglobulin precursor	66932947	163	173.0	29.5	3.28	0.03422
Haptoglobin isoform 1 pre-pro-protein	4826762	45	108.7	14.7	3.79	0.02149
Complement C3 precursor	115298678	187	269.7	31.7	4.32	0.04534
Ceruloplasmin precursor	4557485	122	55.7	6.5	5.14	0.01608
Apolipoprotein A-I pre-pro-protein	4557321	31	40.3	1.7	13.81	0.02004
Carbonic anhydrase 1	192447430	29	29.7	0.7	15.31	0.00834
Peroxiredoxin-2 isoform a	32189392	22	29.7	0.8	18.27	0.00005
Hemoglobin subunit alpha	4504345	15	271.7	3.7	26.52	0.01789
Apolipoprotein B-100 precursor	105990532	516	127.7	0.0	PSC only	0.01591
Hemoglobin subunit delta	4504351	16	26.7	0.0	PSC only	0.00001
Complement C4-B pre-pro-protein	178557739	193	52.3	0.0	PSC only	0.02317
Intercellular adhesion molecule 1 precursor	167466198	58	4.7	0.0	PSC only	0.00097
Peptidyl-prolyl cis-trans isomerase A	10863927	18	5.7	0.0	PSC only	0.00309

MW = molecular weight; SC = spectral count; NSAF = normalized spectral abundance factor.

**Table 6.** Proteins identified as less abundant in cholangiocarcinoma than in primary sclerosing cholangitis

Proteins	Accession	MW (kDa)	Average SC		NSA Fratio	*P*-value (*t*-test)
			CCA	PSC		
Pro-activator polypeptide isoform a pre-pro-protein	11386147	58	0.0	8.0	0.00	0.00496
Trypsin-1 pre-pro-protein	4506145	27	0.0	38.7	0.00	0.01173
Carboxypeptidase B pre-pro-protein	54607080	47	0.0	70.2	0.00	0.02961
Chymotrypsin-like elastase family member 2A pre-pro-protein	15559207	29	0.0	35.5	0.00	0.03517
Chymotrypsin-like elastase family member 3B pre-pro-protein	6679625	29	0.0	43.0	0.00	0.03623
Chymotrypsin-C pre-pro-protein	62526043	29	0.0	21.2	0.00	0.04669
Trefoil factor 2 precursor	4885629	14	1.3	21.0	0.05	0.00235
Antithrombin-III precursor	4502261	53	10.7	43.3	0.18	0.03221
IgG Fc-binding protein precursor	154146262	572	73.7	180.2	0.33	0.00129

## Discussion

Biliary tract and pancreatic malignancies are often diagnosed at advanced stages, with palliative measures being the major option for management of many cases. If diagnosed early, surgical resection or liver transplantation can offer increased survival. The sensitivities of various modalities for diagnosing malignancies in biliary strictures are currently low. We aimed to differentiate benign from malignant biliary strictures by analysing the bile for proteins using LC-MS/MS. We observed that the majority of the proteins identified in bile were similar to those of the plasma (plasma proteins) and certain proteins were differentially expressed among the different groups (CCA, pancreatic cancer, PSC, or benign). Some of those proteins, such as S100 A8 and S100 A9, which were significantly elevated, and TFF-2, which was significantly decreased in malignant strictures in our study, were found to be associated with tumorigenesis.

Serum CA 19-9 has been used as a routine diagnostic and prognostic tool for pancreatic cancer [21, 22]. Detecting malignancies at an earlier stage through lipidomics and proteomics seems promising, and these techniques are in the preliminary stages of being translated into clinical practice. We earlier reported the value of VEGF-1, oxidized phospholipids and volatile organic compounds in malignant biliary strictures and the results appear promising [23–25].

Tissue proteins are altered during the process of carcinogenesis, and this can occur during the earliest stages of malignancy [26]; these proteins can be detected in the extracellular fluid spaces. Detecting these changes in protein/peptide levels in the ECF can increase the diagnostic sensitivity, especially for early lesions. Since bile is in direct contact with biliary tract epithelial cells, malignant transformation of these cells is likely to affect expression of certain proteins in bile. Bile can be a representative of the products of molecular changes in biliary tract epithelia (novel proteins) as it is in direct contact with them. In an earlier study, proteomic analysis of bile fluid revealed that a larger percentage of proteins were cellular, from surrounding organs/tissues [10]. Many proteins associated with cancer, such as S100A9, MUC 1, NGAL, CEACAM6 and several other biomarkers [15, 27, 28] have been identified in bile in patients with malignant biliary strictures, further suggesting the importance of bile in proteomic analysis. Patients with PSC are at an increased risk of developing biliary tract malignancies [29–31], and multiple ERCPs are performed in those patients for relieving the obstruction in the ducts, as well as to rule out malignancies. Also, obtaining bile during routine ERCPs does not impart any major risk to the patients, beyond the baseline risks of the procedure.

We identified several proteins showing differential abundance. Comparing the overall benign (PSC and benign) and malignant lesions (pancreatic cancer and CCA), several proteins including myeloperoxidase, inter-alpha-trypsin inhibitor heavy chain H4, complement C3, ceruloplasmin, alpha-2-macroglobulin, apolipoprotein B-100 and kininogen-1 isoform 2 were found to be significantly elevated in patients with malignant biliary strictures. An earlier study created a model for identification of CCA using proteomic analysis based on 22 peptides, in which they found various proteins—including inter-alpha-trypsin inhibitor heavy chain H4, serum albumin, hemoglobin sub-units alpha and beta, and others—to be significantly elevated in malignant strictures caused by CCA [14]. We observed that similar proteins were also elevated in malignant strictures in our study. These proteins are normally present in bile; however their levels are elevated significantly in the setting of CCA. As in our study, the fact that the above-mentioned proteins were significantly elevated in malignant strictures could be indicative of the changes in protein catabolism/apoptosis taking place in the malignant cells. In another study involving analysis of bile in patients from pancreatic adenocarcinoma, several proteins were identified [10]; of these, proteins such as serum albumin, ceruloplasmin, alpha-2-macroglobulin, vitamin D-binding protein, apolipoprotein A-I were also found to be abundantly expressed in malignant strictures in our study. Our study further confirms the elevation of these above-mentioned proteins in malignant biliary strictures.

Trefoil factor 2 plays an important role in maintaining the integrity of the gut, providing mucosal protection; it has also been found to activate CXCR4 chemokine receptors in epithelial and lymphocytic cancer cell lines, affecting their proliferation [32]. In our study, we found that trefoil factor 2 was significantly lower in the malignant biliary strictures, when compared to the benign ones. Down-regulation of TFF-2, suggestive of tumor invasiveness and metastasis, has been recently described in gastric cancers [33]. We also found that proteins including intercellular adhesion molecule 1, ceruloplasmin, haptoglobulin, complement c3, and -c4b were found to be significantly elevated in CCA when compared to patients with PSC. However, considering that only three patients in our study had CCA, the clinical significance of this observation warrants further validation from future studies. The S100 group of proteins, which are especially the markers of schwannomas, have been linked with many malignant conditions [34–36]. A significant elevation of S100 A9 in bile in patients has been demonstrated in CCA [27]. In our study, S100 A8, S100 A9 and a few other biomarkers were significantly elevated in pancreatic cancer, when compared with benign lesions and PSC.

Although the complex constitution of bile can limit proteomic analysis, recent improvements in technology have facilitated major advances in proteomic studies. Summarizing, proteomic analysis of bile reflects the cellular changes in the surrounding tissues/organs, which can be identified through differential expression of various peptides/protein markers. Our preliminary study, involving quantitative proteomic analysis of 24 patients with biliary strictures, shows that the protein biomarkers may differentiate malignant- from benign biliary strictures and may at an earlier stage detect malignancies (CCA) in PSC patients with indeterminate biliary strictures, and hence improve their prognoses. Future large-scale studies on proteomic analysis of bile, comparing benign and malignant strictures, will further enhance the results and its application in clinical practice.
